# Adsorption and controlled release of three kinds of flavors on UiO‐66

**DOI:** 10.1002/fsn3.1477

**Published:** 2020-02-24

**Authors:** Deshou Mao, Congjia Xie, Zhiyu Li, Liu Hong, Rongfen Qu, You Gao, Jiao He, Jiaqiang Wang

**Affiliations:** ^1^ Research & Technology Center of Yunnan Industrial of China Tobacco Industry CO., Ltd Kunming China; ^2^ National Center for International Research on Photoelectric and Energy Materials Yunnan Province Engineering Research Center of Photocatalytic Treatment of Industrial Wastewater Yunnan Provincial Collaborative Innovation Center of Green Chemistry for Lignite Energy School of Chemical Sciences & Technology Yunnan University Kunming China

**Keywords:** adsorption and controlled release, eugenol, isophorone, UiO‐66, β‐ionone

## Abstract

Delivery systems for controlled release of fragrances are significantly essential in the flavor and fragrance industry due to a limited life span (premature evaporation and degradation) of fragrance compounds. Recently, several adsorption materials such as porous materials have been developed in delivery systems for targeted fragrance release. In this work, UiO‐66, a member of metal–organic framework (MOF) family with high porosity and greater adsorbability, was selected as a prospective alternative to traditional porous adsorbents for controlled release of fragrances. Isophorone, eugenol, and β‐ionone with strong aroma are widely used as perfume flavors, soap flavor, cosmetic flavors, and even as a food‐flavoring agents, and were chosen as representative fragrances for adsorption and controlled release studies. The adsorption and release behavior of fragrances on UiO‐66 was evaluated by high‐performance liquid chromatography (HPLC). The UiO‐66 with high surface area (1,076 m^2^/g) achieved effective storage and controlled release for isophorone, eugenol, and β‐ionone. The adsorption rates of isophorone, eugenol, and β‐ionone can reach 99.4%, 99.9%, and 60.2%, respectively. Additionally, the release of these fragrances from UiO‐66 can sustain over 20 days. UiO‐66 exhibited higher release rate over eugenol with desorption rates of 95.2% than that of β‐ionone (52.6%) and isophorone (49.6%), respectively, suggesting a good adsorption‐release selectivity of UiO‐66 to different fragrances. This study further confirms the usability of UiO‐66 in fragrance release and extends the application of MOF porosity in aroma release.

## Graphical abstract

The micropore‐dominated Zr‐based metal framework (UiO‐66) with high surface area (1,076 m^2^/g) exhibits excellent adsorption capacity (60.2%~99.9% of adsorption rate) and effective controlled release (49.6%~95.0% release rate) over 20 days toward common flavors (isophorone, eugenol, and β‐ionone), which can be considered as a novel prospective porous material for controlled release of fragrances due to more advantages than traditional porous materials. The adsorption and diffusion mechanism of flavor substances on UiO‐66 was revealed.

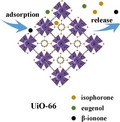

## 1 INTRODUCTION

The fragrance (Levy et al., 2017; Veith, Perren, & Pratsinis, 2005) with characteristic scents and antibacterial properties has been widely used in the fields of medical products (Bhattacharjee, Gumma, & Purkait, 2018), daily chemicals (Ghayempour & Montazer, 2016), foods (Siahaan, Pendleton, Wooc, & Chun, 2014), and cosmetics (Erucar & Keskin, 2016) to enhance the quality and characteristic flavor of the products. Generally, fragrant molecules are organic compounds with specific functional groups such as aldehyde (Levy et al., 2017), ketone (Kuhnt, Herrmann, Benczédi, Foster, & Weder, 2015), and acid (Veith et al., 2005). As a class of highly volatile compounds, spices can be quickly perceived by the human body, while they generally lose their aroma rapidly causing poor durability in final products (Golja, Sumig, & Tavcer, 2013). Currently, the delivery system has been widely employed to capture these volatile molecules and achieve controlled release of fragrances (Liu, Chen, Fishman, & Hicks, 2005). Porous materials (Kaura, Kukkar, Bhardwaj, Kim, & Deep, 2018) such as zeolites (Tekin & Bac, 2016), silica nanoparticles (Hu, Liu, Xie, & Wu, 2013), activated carbon (Fernandez‐Perez, Villafranca‐Sanchez, Flores‐Cespedes, Garrido‐Herrera, & Perez‐Garcia, 2005), aluminosilicate (Ghodke, Sonawane, Bhanvase, Mishra, & Joshi, 2015), and calcium carbonate (Levy et al., 2017) have been used extensively as supporters or carriers for the delivery release of various kinds of fragrances. They exhibit well storage and release capacity which depends upon the properties of pore structure, molecular structure, and the interaction of fragrance molecules with porous materials (Ganicz, Kurjata, Perry, & Stanczyk, 2015; Ghayempour & Montazer, 2016). However, most delivery systems always suffer from the problems such as early evaporation and degradation of flavors on scaffold materials during adsorption process (Madene, Jacquot, Scher, & Desobry, 2006), vulnerable to external factors (temperature, humidity, or chemical interactions; Cao et al., 2015; Ghodke et al., 2015), or supporters with strong adsorption capacity but weak desorption (Ciriminna & Pagliaro, 2013), or vice versa.

Metal–organic frameworks (MOFs; He, Chen, Lü, & Liu, 2014; Lin, Liu, & Chen, 2016; Qiu, Feng, Zhang, Jia, & Yao, 2017) are an emergent class of porous materials consist of organic–inorganic structure, which shows more advantages than traditional support materials due to their unique hybrid structures (high surface area and porosity) and relatively milder synthesized conditions (in terms of solvent, pH, and temperature; Li et al., 2014; Mu, Jiang, Chao, Lou, & Chen, 2018; Yang et al., 2018). First, MOFs are compositionally and structurally diverse, allowing for the facile synthesis of MOFs of different compositions, shapes, sizes, and chemical properties. Second, MOFs are intrinsically biodegradable as a result of relatively labile metal–ligand bonds, making it possible to rapidly degrade and clear the nanocarriers after the intended task is completed (Rocca, Liu, & Lin, 2011). Though the moderately low chemical and aqueous stability of MOFs has limited their scope for industrial applications, this drawback of MOFs is considered an advantage for delivery system in light industry or medicine applications, as the MOF particles can be biodegraded and eliminated from the body after the compounds are released (Orellana‐Tavra et al., 2016). At present, there are some studies of MOFs used as nanocarriers for drug delivery (ibuprofen [Erucar & Keskin, 2016], alendronate [Golmohamadpour, Bahramian, Shafiee, & Mamani, 2018], doxorubicin hydrochloride [Bhattacharjee et al., 2018]), VOC removal (Xu et al., 2018; Zhang, Lv, Shi, Yang, & Yang, 2019; Zhu, Hu, Tong, Zhao, & Zhao, 2017), and sustained release of small molecule dyes (Li et al., 2016) and cosmetic molecules (caffeine and urea; Erucar & Keskin, 2016). With recent success in assembling edible MOFs (CD‐MOFs) using γ‐cyclodextrin and potassium ion (Smaldone et al., 2010), it is expected that MOFs could be applied in the food industries in the near future (Wu et al., 2019). Most recently, MOFs have been investigated as novel allyl isothiocyanate (AITC) carrier for food safety and food industry applications, which allow the successful implementation of AITC into food systems by improving its stability while eliminating or reducing its negative organoleptic impact on various food matrices (Wang et al., 2016). One well‐recognized challenge of MOFs is their poor aqueous stability, limiting their scope for practical applications. However, this drawback of MOFs is considered an advantage for delivery system in food industry or biomedicine applications, as the MOF particles can be biodegraded and eliminated from the body after the volatile compounds are released (Orellana‐Tavra et al., 2016).

Among various MOFs, Zr‐based MOFs UiO‐66 exhibits a significant advantage compared with many other MOFs such as Cu‐, Fe‐, and Cr‐based MOFs (Lashkari, Wang, Liu, Li, & Yam, 2017) owing to its interesting thermal and chemical stabilities. UiO‐66 has been widely used as an adsorbent or carrier in the fields of release system as well. Cunha et al. (2013) demonstrated that UiO‐66 presented efficient performance for encapsulation and release of cosmetic caffeine within 24 hr among a series of biocompatible MOFs (MIL‐100, UiO‐66, MIL‐127, and MIL‐53), which still suffer from a relatively low loading capacity of 65.8% and rapid complete release within 0.5 hr. Moreira et al. (2012) investigated the selective adsorption–separation of xylene isomers in the liquid phase using Zr‐based MOF UiO‐66 exhibiting selectivity values for m‐xylene (1.8) and p‐xylene (2.4) at 313 K in the presence of n‐heptane.

Based on the above studies, herein, we show for the first time that UiO‐66 as a carrier for adsorption and controlled release of model flavor compounds (isophorone, eugenol, and β‐ionone). This work aims to develop an efficient adsorbent for better adsorption capabilities and more controllable release of simple fragrance molecules. The properties of nanocarrier system were confirmed by BET surface analysis, scanning electron microscope (SEM), X‐ray powder diffraction (XRD), Fourier transform infrared (FTIR), and thermogravimetric analysis (TGA). As expected, the prepared UiO‐66 exhibited efficient adsorption and achieved selective long‐lasting (over 20 days) release toward representative flavors, demonstrated a novel supporter in aroma release system, expanding the applications of nanoMOF in fragrance industries. Finally, the relationships of material's structural properties and adsorption–desorption performance were discussed.

## 2 MATERIALS AND EXPERIMENTAL

### 2.1 Materials

Zirconium chloride (ZrCl_4_, >98%) and 1,4‐benzendicarboxylic acid (H_2_BDC, >98%) were purchased from Adamas Reagent Co., Ltd. N, N‐dimethylformamide (DMF, AR) and anhydrous ethanol (AR) were purchased from Guangdong Guanghua Sci‐Tech Co., Ltd. Cyclohexane (AR) was obtained from Tianjin Fengchuan Chemical Reagent Technologies Co., Ltd. Isophorone, eugenol, and β‐ionone (>98%) were provided by Yunnan Industrial of China Tobacco Industry Co., Ltd. The physical and chemical properties of the fragrance compounds are shown in Table S1.

### 2.2 Preparation of UiO‐66

The synthesis of UiO‐66 followed the procedure reported by Silva, Luz, Xamena, Corma, and Garcia (2010) with slight modification. 0.625 g of ZrCl_4_ and 0.615 g of 1,4‐benzenedicarboxylic acid (H_2_BDC) were dissolved in 50 ml dimethylformamide (DMF). The solution was transferred into a stainless steel autoclave with Teflon‐lined and heated at 120°C for 24 hr. When the autoclave was naturally cooled to room temperature, the solid was filtered out and washed with ethyl alcohol and then dried at 90°C. The dried product was impregnated with DMF for 6 hr, then filtered, washed three times with ethanol, and dried at 90°C. The solid was then transferred into a stainless steel autoclave with Teflon‐lined and alcoholized with ethanol at 90°C for 5 hr, centrifuged, and washed with ethanol and acetone to obtain a solid product. The final product was dried at 90°C.

### 2.3 Characterizations

Nitrogen adsorption–desorption was taken on a NOVA 2000e gas sorption analyzer (Quantachrome Corp.) to determine the pore‐size distributions, BET surface areas, and pore volumes. N_2_ sorption isotherms were obtained at 77 K using a Micromeritics ASAP 2460. Thirty milligram of the sample was activated at 140°C under primary vacuum overnight. The total pore volume and pore‐size distribution were evaluated by the NLDFT (nonlocal density functional theory) method from the adsorption branch of the isotherm and using a cylindrical model. XRD experiments were conducted on a D/max‐3B spectrometer with Cu Kα radiation, and scans were made in the 2θ range 0.1–5° with a scan rate 0.05°/min (low‐angle diffraction), and in the 2θ range 5–90° with a scan rate of 10°/min (wide‐angle diffraction). SEM analysis was taken on a FEIQuanta200FEG microscope with an accelerating voltage of 15 kV to observe the morphology of UiO‐66. FTIR spectra were recorded using a Thermo Nicolet 8700 instrument in the range of 4,000–400 cm^−1^. TGAs were carried out on a Mettler Toledo TGA/DSC/1600LF in temperature range 25–800°C with a 10°C/min heating rate under an N_2_ atmosphere (20 ml/min).

### 2.4 Quantitative evaluation by HPLC

Considering the accurate quantitative analysis of low concentration of fragrance solution, HPLC was selected to determine all fragrance solution concentrations calculated by corresponding peak areas. HPLC analysis was conducted on an Agilent 1260 Infinity instrument at 30°C with a 150 × 4.6 mm column (5 μm particles). The flow rate was 1.0 ml/min; mobile phase, methanol: water (40:60, v/v); the detection wavelength, 232 nm; the injection volume, 10 μl; and retention time, 9 min. Different concentrations of fragrances in ethanol (10, 20, 50, 100, and 150 mg/ml) were analyzed by HPLC under above conditions to compute the standard curves (peak areas vs. fragrance concentrations) which are shown in Figures S1–S3 and Tables S2–S4. The correlation coefficients (*r*) of isophorone, eugenol, and β‐ionone were .9992, .9989, and .9999, respectively, availed in the following adsorption and release experiments.

### 2.5 Adsorption of fragrances in UiO‐66

The adsorption experiments of UiO‐66 toward isophorone, eugenol, and β‐ionone were carried out at room temperature. Typically, 100 ppm of spice solution was dispersed in 15 ml of solvents (ethanol and cyclohexane). Subsequently, different amounts (3–30 mg) of UiO‐66 were added to the solution with stirring for 3 hr. Aliquots (1 ml) from the mixed solution were taken in certain time intervals (10 min), and the intervals were adjusted to lager intervals (1 hr) after 1 hr. The clear solution was obtained by filtration using a 0.22‐μm filter, and then further analyzed by HPLC to evaluate the adsorption capacity of UiO‐66 toward fragrances. The fragrance adsorption rates were calculated using the following equations:

(1) $$\text{Adsorption rate}\;\left(\%\right)=\left(C_{0}-C_{t}\right)/C_{0}\times 100$$

where *C*_0_ and *C_t_* represent the fragrance concentrations (mg/L) at initial time *t*, respectively.

### 2.6 Controlled release of fragrances in UiO‐66

One hundred fifty milligram of UiO‐66 was dispersed into 150 ml of cyclohexane solution containing 100 ppm of fragrances with stirring for 2 hr, filtered, and naturally dried to obtain the precipitation. Aliquots (10 mg) of the solid precipitation were taken and redispersed into 5 ml of ethanol solution in certain time intervals (1 hr), and the intervals were adjusted to lager intervals (24 hr) after 24 hr. The mixed solution was centrifuged and washed with 5 ml of ethanol for three times, and then, 15 ml of supernatant liquid was totally obtained and filtered with a needle (2.2 μm) and then examined by HPLC. The released capacity of UiO‐66 was evaluated by remained fragrance amount or percent remained fragrance in adsorbent after elution calculated as follows:

(2) $$\text{Remained fragrance}\text{amount}\left(\text{mg}/\text{g in adsorbent}\right)=\left(M_{0}-M_{f}\right)/M$$

(3) $$\text{Percent remained fragrance}\left(\%\right)=\left(M_{0}-M_{f}\right)/M\times 100$$

where *M*_0_ (mg) is the initial mass of fragrances in the solution and *M*_f_ (mg) is the mass of released fragrances in the final supernatant. *M* (g) represents the mass of the UiO‐66 before loading fragrances.

## 3 RESULTS AND DISCUSSIONS

### 3.1 Characterizations of UiO‐66

The structural properties of obtained UiO‐66 were examined by N_2_ adsorption–desorption, powder X‐ray diffraction (XRD), and scanning electron microscopy (SEM), and the results were summarized in the supplementary. The XRD pattern of UiO‐66 is shown in Figure S4. The characteristic diffraction peaks appeared in 2θ between 3° and 60°, which were consistent with that in reported XRD pattern (Abid et al., 2012). The adsorption–desorption isotherm (Figure S5a) and pore‐size distribution (Figure S5b) of UiO‐66 implied that UiO‐66 exhibited a typical type I isotherm (IUPAC classification), dominated by a large number of micropores range from 2 to 10 Å. The BET surface area, pore volume, and pore size of UiO‐66 (Table S5) are 1,076 m^2^/g, 0.12 cm^3^/g, and 6.7 nm, respectively. The SEM images in Figure S6a,b confirmed that as‐prepared UiO‐66 is uniform intergrown particles and easily agglomerated without specific shape, corresponding to that reported by Lv, Liu, Xiong, Zhang, and Guan (2016). All these results demonstrated that the UiO‐66 was successfully synthesized.

In order to explore the potential influence of the fragrances loading on the structure of UiO‐66, a series of characterization of PXRD (Figure S4), FTIR (Figure S7), and TGA (Figure S8) were performed for UiO‐66 and fragrance‐containing UiO‐66. Apparently, XRD patterns in Figure S4 shows that there is no significant change in domain UiO‐66 crystalline structure after the fragrance (isophorone, eugenol, and β‐ionone) adsorption and after release for 20 days. The FTIR spectra of UiO‐66 in Figure S7 show characteristic bands corresponding to carboxylate (‐O‐C‐O‐) groups at 1,400 and 1,584 cm^−1^ and the peak at 3,400 cm^−1^ corresponding to the free water molecules in the pores. All absorption peaks correlate well with the literature report (Li et al., 2016). The signal is less intense for isophorone/UiO‐66 (Zr) compared with that of pure UiO‐66, while the signal intensity of β‐ionone/UiO‐66 and eugenol/UiO‐66 was higher than that of pure UiO‐66, respectively. This deviation is most probably due to the fragrance adsorption on UiO‐66 result in the change in the dipoles. The TGA curve in Figure S5 shows three stages of weight losses for as‐synthesized materials which can be attributed to water departure (25–130°C), DMF or fragrance departure (150–400°C), and decomposition of ligand and collapse of UiO‐66 structure (400–650°C). Meanwhile, UiO‐66 after fragrance adsorption presents lower water loss, and the eugenol/UiO‐66 further exhibited less ligand decomposition, suggesting that more or less fragrance adsorption on the surface of UiO‐66 reduces the surface water evaporation, resulting in higher thermal stability of Zr‐MOF.

All these results demonstrate the successful construction of fragrance/UiO‐66. This indicates that the crystalline structure of UiO‐66 was highly stable during adsorption process and remained intact in contact with fragrances, suggesting the promising potential of UiO‐66 in fragrance adsorption and release applications.

### 3.2 Effect of operation parameters on adsorption

#### 3.2.1 Effect of solvents

**Figure 1:**
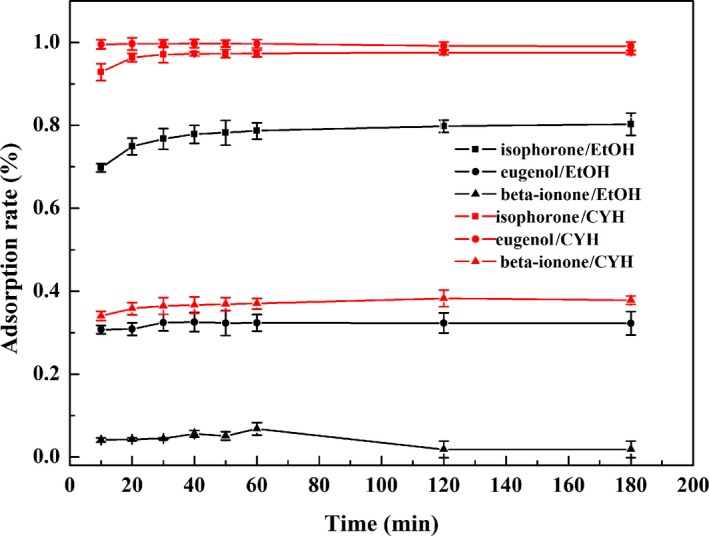
Adsorption curves of UiO‐66 for three fragrances in ethanol solutions and cyclohexane solutions

Isophorone, eugenol, and β‐ionone were selected as typical fragrances to investigate the effect of solvents on adsorption process. Figure 1 presents the adsorption capacity of UiO‐66 toward isophorone, eugenol, and β‐ionone in ethanol or cyclohexane solvent within 180 min. It is evident that different solvents had significant influence on fragrance adsorption. All fragrances suffered great decrease in adsorption in ethanol solution than in cyclohexane solution, which may be attributed to a more polarity of ethanol solvent (Fang et al., 2017) and the formation of hydrogen bond between ethanol solvent and fragrance molecules causing a larger adsorption‐free energy and a lower adsorption capacity. As illustrated in Figure 1, all adsorption behavior displayed a similar trend that increased in the first stage and then achieved a balance. In cyclohexane solution, the adsorption equilibrium of isophorone, eugenol, and β‐ionone on UiO‐66 was approached at 30, 10, and 30 min, respectively. In addition, UiO‐66 exhibited high adsorption to isophorone and eugenol with adsorption rates of 97.6% and 99.8%, respectively. Comparatively, the adsorption rate of β‐ionone was much lower (38.3%) due to space steric hindrance resulted by lateral chains of β‐ionone during the adsorption process.

#### 3.2.2 Effect of adsorbent dosage

**Figure 2:**
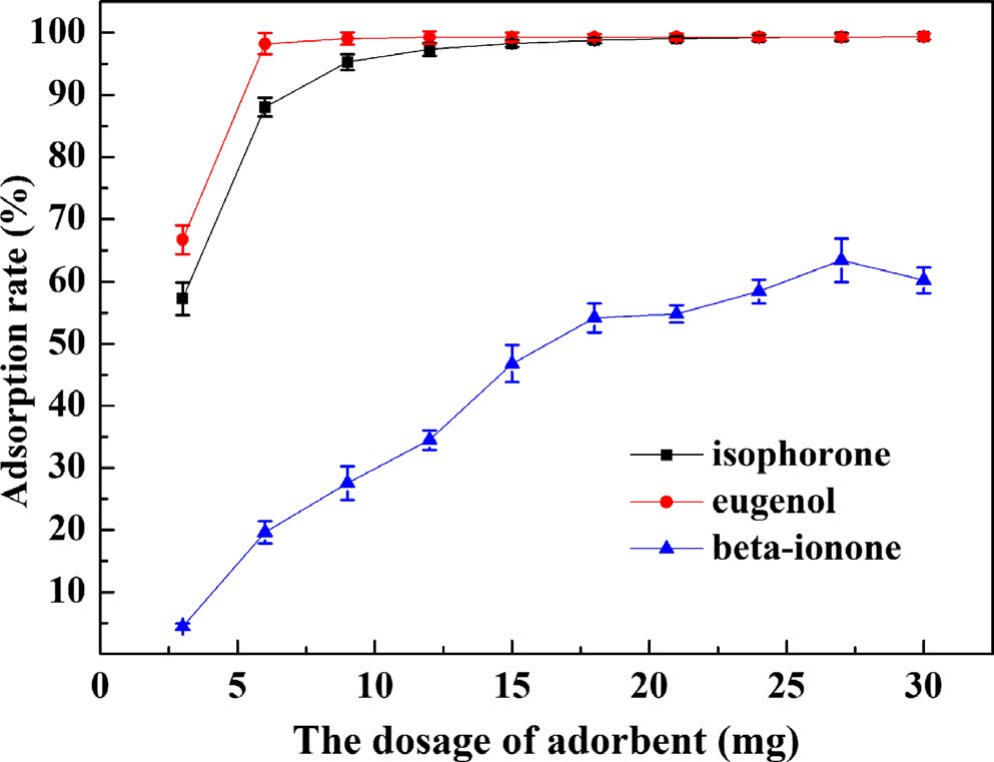
Effect of adsorbent dose on adsorption of isophorone, eugenol, and β‐ionone in cyclohexane solution

The effect of adsorbent dosage on fragrance adsorption in cyclohexane solution was investigated at pH 7 and the initial fragrance concentration of 100 ppm. As shown in Figure 2, with an increase in dose from 3 to 30 mg, the adsorption rate of isophorone, eugenol, and β‐ionone significantly increased from 57.2% to 99.4%, 66.7% to 99.9%, and 44.4% to 60.2%, respectively, due to the increase in surface area and active sites enhancing the adsorption capacity. Nevertheless, the adsorption rate of isophorone, eugenol, and β‐ionone reached maximum at 9, 6, and 18 mg of UiO‐66, respectively, and then, no further increase in adsorption rate is observed after that on account of the decrease in the adsorption sites. Therefore, the optimum dose of UiO‐66 was fixed as 9, 6, and 18 mg, respectively.

### 3.3 Controlled release study of fragrances on UiO‐66

#### 3.3.1 Release capacity under different temperatures

**Figure 3:**
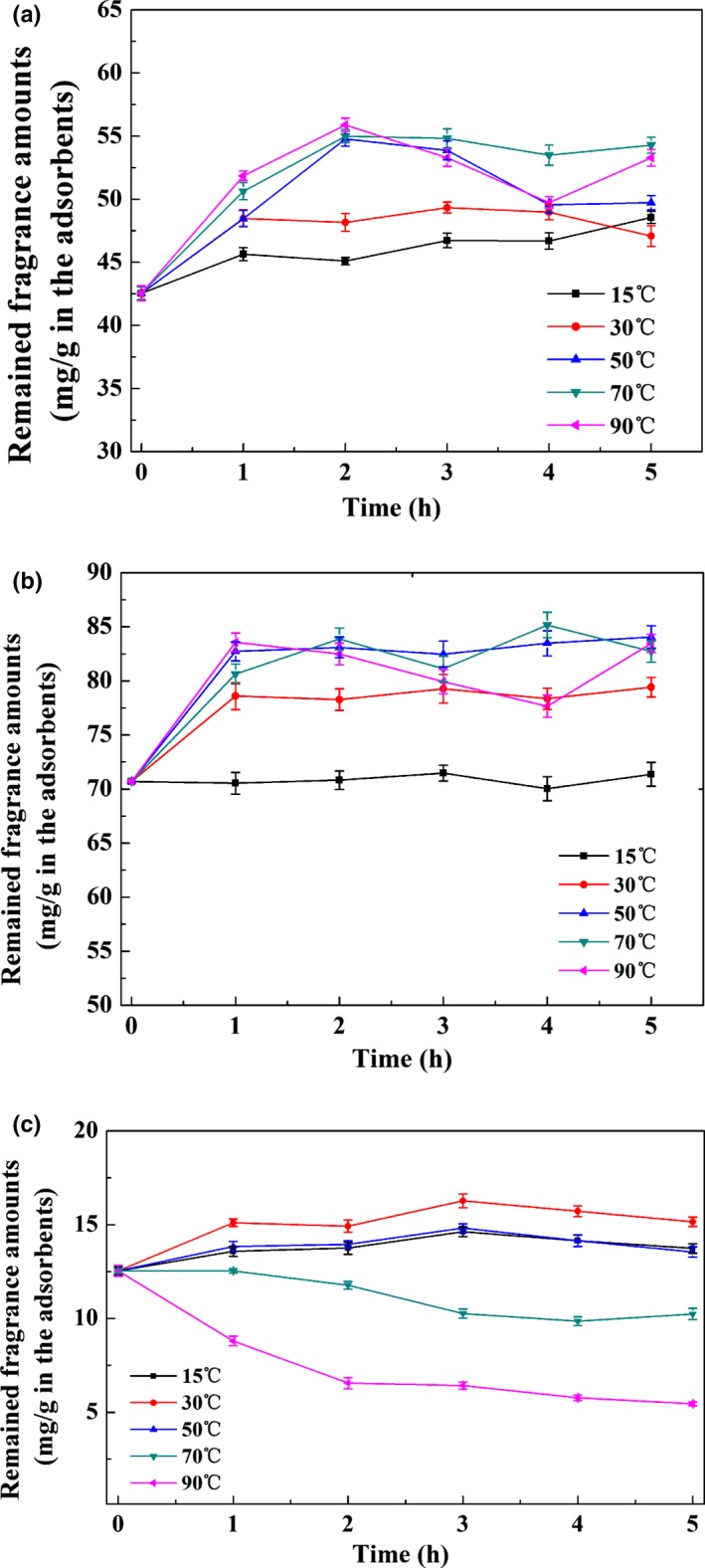
(a) Controlled release curve of isophorone from UiO‐66 at different temperatures. (b) Controlled release curve of eugenol from UiO‐66 at different temperatures. (c) Sustained release curve of β‐ionone from UiO‐66 at different temperatures

The effect of different temperatures on release behavior of volatile fragrances from UiO‐66 was investigated. The sustained release of isophorone, eugenol, and β‐ionone from UiO‐66 was evaluated by measuring the loading amount (fragrance remained in adsorbent mg/g). Figure 3a–c shows the release profile of fragrances from UiO‐66 at different temperatures (15–90°C) within 5 hr. The loading amount of isophorone and eugenol on UiO‐66 increased sharply and then slowed down with the increase in temperature, which were from 48.6 to 53.3 mg/g and 71.4 to 83.4 mg/g, respectively, indicating a reduced release rate. It may be because the molecular motion accelerated with the increase in temperature, and the fragrances initially adsorbed in relative large pores can diffuse into smaller micropores. After the diffusion reached a stable equilibrium, the solid loading reached a stable value, whereas for β‐ionone in Figure 3c, the loading amount of β‐ionone on UiO‐66 at 30°C with 15.2 mg/g was higher than that at 15°C with 13.7 mg/g, exhibiting slight decrease in release rate. Then, the delivery system displayed an obviously increasing release rate and decreasing loading amount with the increase in temperature from 30 to 90°C, which may be because the movement energy of molecules is greater than the weak surface adsorption energy at high temperature, causing a vast majority of β‐ionone adsorbed on the surface of UiO‐66 could be released. In summary, temperature acted as a kind of activator for β‐ionone release, so that the cumulative release of β‐ionone (from 13.7 to 5.4 mg/g) was 60.6% during the temperature range.

#### 3.3.2 Long‐time release study

**Figure 4:**
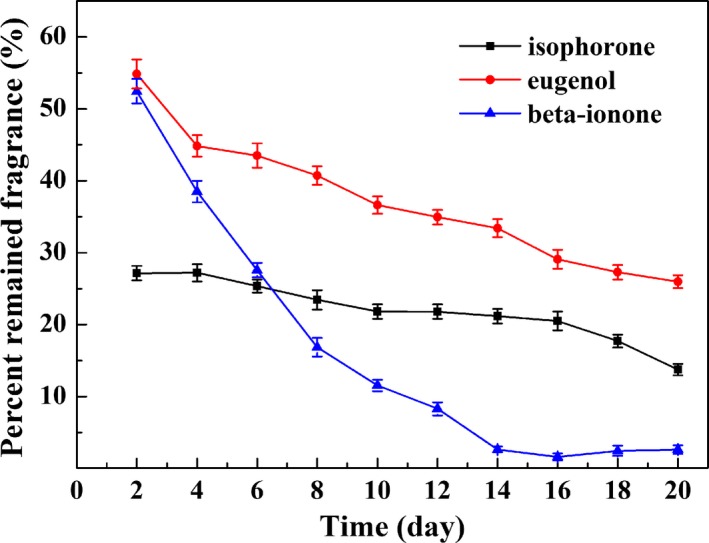
Release of fragrances from UiO‐66 at room temperature for 20 days

**Figure 5:**
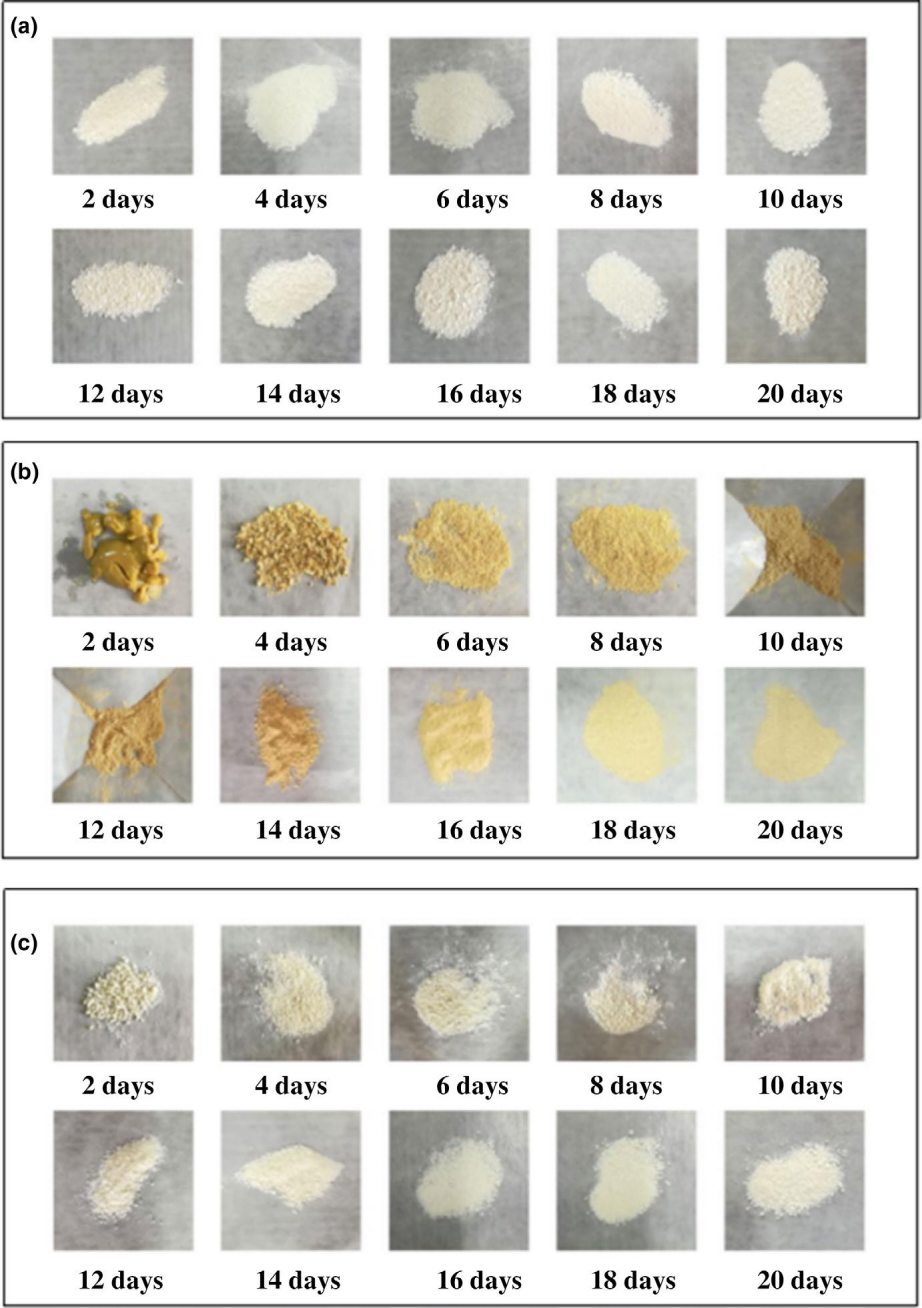
(a) The corresponding state variation of isophorone loaded UiO‐66 during 20 days of release. (b) The corresponding state variation of eugenol loaded UiO‐66 during 20 days of release. (c) The corresponding state variation of β‐ionone loaded UiO‐66 during 20 days of release

Figure 4 shows the controlled release capacity of the fragrances at room temperature for 20 days, while Figure 5 correspondingly shows the variation of UiO‐66 loaded with fragrances during the controlled release process. The loading amount of eugenol and β‐ionone on UiO‐66 reduced sharply within 14 and 16 days, respectively, and then reduced slowly afterward, which were reduced from 54.8% to 2.6% and 52.4% to 26.3%, respectively. Meanwhile, the fragrance‐loaded adsorbents from Figure 5a,b undergo a significant change during sustained release, gradually varying from viscous to loose. It was due to the decrease in diffusion rate of fragrance with the decrease in the concentration of flavor molecules. However, there was no obvious release of isophorone on UiO‐66 with loading amount from 27.2% to 13.7% within 20 days and the samples (Figure 5c) retained steady state of loose during the sustained release process. The results indicate that the release of eugenol and β‐ionone from UiO‐66 is more controlled and effective than that of isophorone. These flavors and fragrances can be slowly released over a long period of time. The order of desorption rate from the adsorbent is β‐ionone with 95.2% > eugenol with 52.6% > isophorone with 49.6%.

### 3.4 Possible adsorption and controlled released mechanism

**Figure 6:**
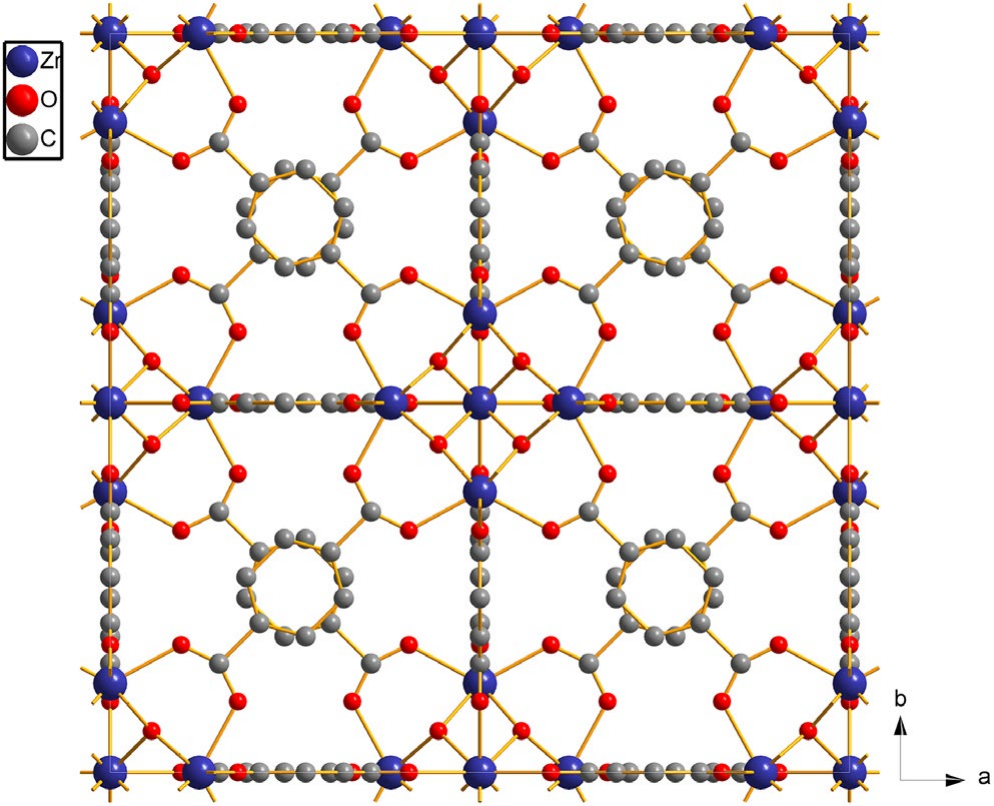
Chemical structures of UiO‐66 in two‐dimensional (2D) view (Zr: blue, O: red, C: gray)

**Figure 7:**
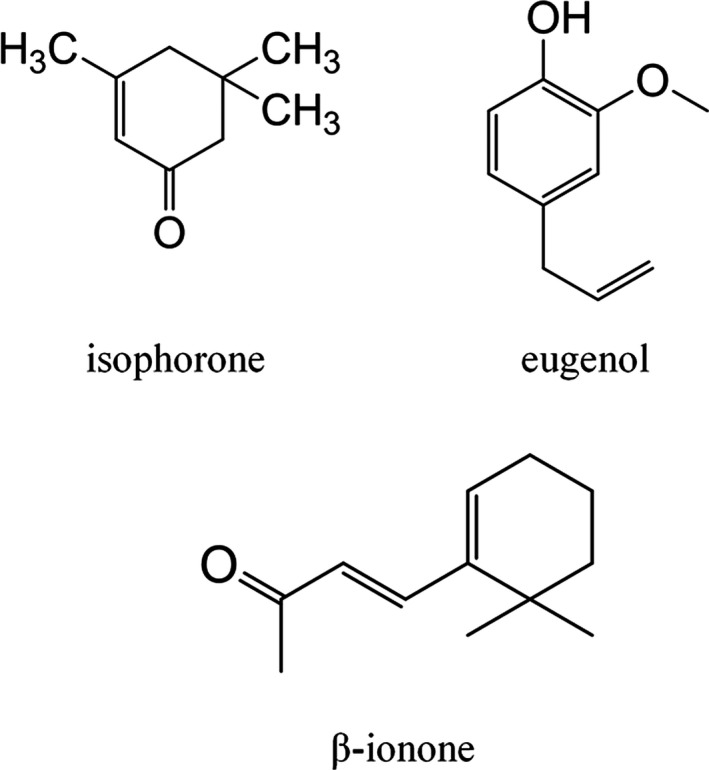
Chemical structures of fragrances in this work (isophorone, eugenol, β‐ionone)

As demonstrated in N_2_ adsorption–desorption analysis, the sample UiO‐66 (Figure 6) has small pore sizes range from 2 to 10 Å. The micromolecular and simple fragrances (Figure 7) can be adsorbed inner the pores or on the surface of UiO‐66. The adsorption of fragrance molecules by adsorbents mainly belongs to physical adsorption and is mainly affected by the pore structure of adsorbents, structure of fragrance molecules, and interactions (hydrogen bonding and π‐π interaction). The high specific surface area of UiO‐66 can exhibit high adsorption capacity to the fragrance molecules. The adsorption amount of eugenol was the largest probably due to the hydrogen bonding and π‐π interaction between the eugenol molecules and UiO‐66. The lowest adsorption capacity of β‐ionone may be attributed to its large molecular size and long branch occupying many adsorption sites during adsorption process. The smallest molecular size of isophorone can easily enter into the interior of the pores resulting in a relatively high adsorption capacity on UiO‐66.

Based on the studies (Zhou et al., 2017), the desorption and release of fragrances on porous materials undergoes general diffusion, Knudsen diffusion, and surface diffusion. The higher the proportion of micropores in adsorbents is, the higher the proportion of surface diffusion in the release process is, and the better effect the sustained release can achieve. Therefore, UiO‐66, mainly microporous structure, can be regarded as desirable porous material for sustained release. The difference in sustained release effect is similarly related to the structure of fragrance molecules and the interaction between the adsorbent and the fragrances. When the bulky fragrances accumulated on the surface of the material, rapid diffusion and desorption can be achieved during the sustained release process. On the other hand, small molecular size fragrances retained in interior of the pores of the material after adsorption and were not easily volatilized and released resulting in undesirable sustained release capacity. The interaction between fragrance and adsorbents (hydrogen bonding and π‐π interaction) will increase the diffusion resistance of fragrance causing a relatively slower release.

## 4 CONCLUSION

As confirmed, UiO‐66 in our work can be used for effective adsorption and controlled release of commonly used fragrances of isophorone, eugenol, and β‐ionone. Different solvents and adsorbent dosage had a great influence on the adsorption process. In cyclohexane solutions, the maximum adsorption rates of UiO‐66 toward isophorone, eugenol, and β‐ionone were 99.4%, 99.9%, and 60.2%, respectively, rapidly approached at 30, 10, and 30 min, respectively. The results of slow release indicate that these flavors and fragrances can be slowly released over a long period of time. Particularly, the release of eugenol and β‐ionone from UiO‐66 is more controlled and effective than that of isophorone. The order of desorption rate from the adsorbent is β‐ionone with 95.2% > eugenol with 52.6% > isophorone with 49.6%. In addition, temperature had different influence on release behavior of these fragrances. Increasing temperature accelerates the adsorption of isophorone, eugenol converted to chemical adsorption, advertising to their slow release, while temperature rise will avail to the release of β‐ionone from UiO‐66.

## CONFLICT OF INTEREST

The authors declare that they do not have any conflict of interest.

## ETHICAL APPROVAL

This study does not involve any human or animal testing.
