# Supplementary material for: Adsorption and controlled release of three kinds of flavors on UiO‐66
Source: Food Sci Nutr. 2020 Feb 24;8(4):1914–22. doi: 10.1002/fsn3.1477 (PMC7174235; doi:10.1002/fsn3.1477)
Supplement: Supplementary file 1 [file FSN3-8-1914-s001.docx]

Supplementary Information

**Adsorption and Controlled Release of Three Kinds of Flavors on UiO-66**

Deshou Mao ^1^, Congjia Xie ^2^, Zhiyu Li ^1^*, Liu Hong ^1^, Rongfen Qu ^1^, You Gao ^2^, Jiao He ^2^, and Jiaqiang Wang ^2^*

1. Research & Technology Center of Yunnan Industrial of China Tobacco Industry CO., Ltd, Kunming, 650231, P.R. China;
2. National Center for International Research on Photoelectric and Energy Materials, Yunnan Province Engineering Research Center of Photocatalytic Treatment of Industrial Wastewater, Yunnan Provincial Collaborative Innovation Center of Green Chemistry for Lignite Energy, School of Chemical Sciences & Technology, School of Energy, Yunnan University, Kunming 650091, P.R. China.

* Correspondence: jqwang@ynu.edu.cn (J. Wang) or 1379317701@qq.com (Z. Li)

Table S1. The physical and chemical properties of the fragrance compounds.

| Compounds | Purity | Structural Formulas | Molecular Weights | Density  (g/mL,20℃) |
| --- | --- | --- | --- | --- |
| isophorone | ≥98% |  | 138.21 | 0.905 |
| eugenol | ≥98% |  | 164.20 | 1.066 |
| β-ionone | ≥98% |  | 192.30 | 0.945 |

Table S2. Peak areas of different concentrations of isophorone detected by HPLC

| c (ppm) | 10 | 20 | 50 | 100 | 150 |
| --- | --- | --- | --- | --- | --- |
| A | 412.5 | 809.4 | 1979.3 | 3923.1 | 5673.7 |

Fig. S1 standard curve of isophorone

Note: The standard curve equation of isophorone: A=37.8*c+67.9, R^2^=0.9992

Table S3. Peak areas of different concentrations of eugenol detected by HPLC

| c (ppm) | 10 | 20 | 50 | 100 | 150 |
| --- | --- | --- | --- | --- | --- |
| A  | 350.9 | 730.1 | 1713.5 | 3422.8 | 5329.8 |

Fig. S2 standard curve of eugenol

Note: The standard curve equation of eugenol: A=35.2*c-16.1, R^2^=0.9989

Table S4. Peak areas of different concentrations of β-ionone detected by HPLC

| c (ppm) | 10 | 20 | 50 | 100 | 150 |
| --- | --- | --- | --- | --- | --- |
| A | 279.7 | 555.9 | 1434.6 | 2865.6 | 4278 |

Fig. S3 standard curve of β-ionone

Note: The standard curve equation of β-ionone: A=28.6*c-5.8，R^2^=0.9999


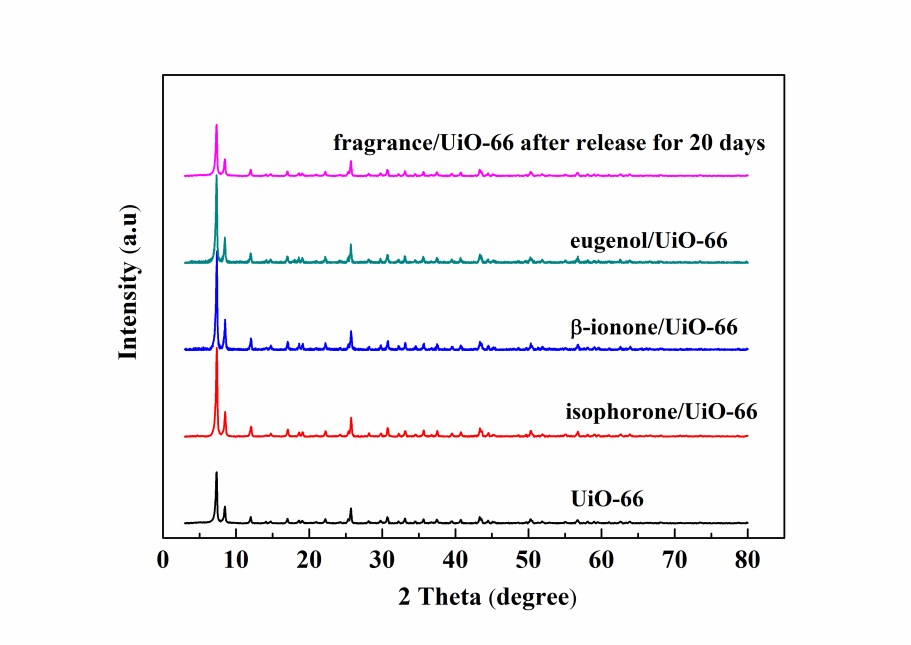


Fig. S4 XRD patterns of empty UiO-66(Zr), fragrances containing UiO-66(Zr) and fragrances/UiO-66(Zr) after release for 20 days.


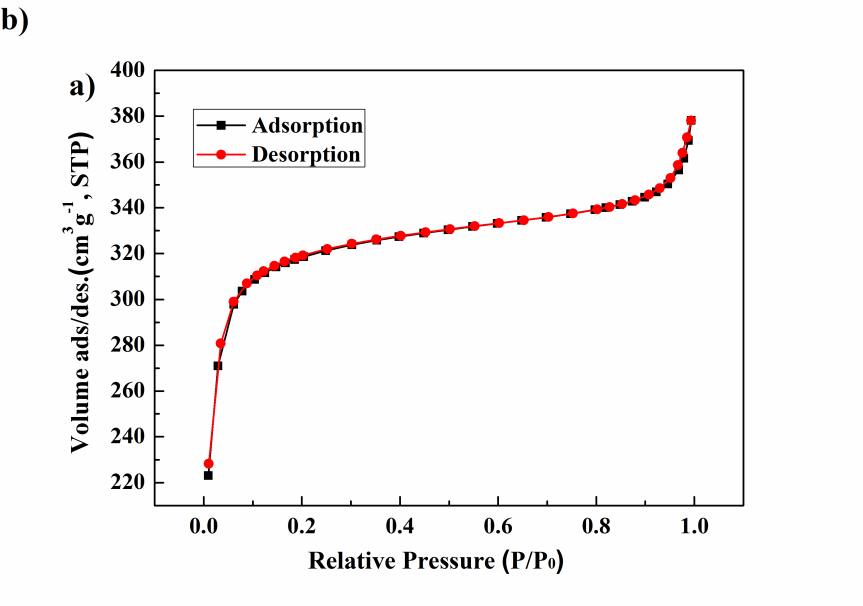


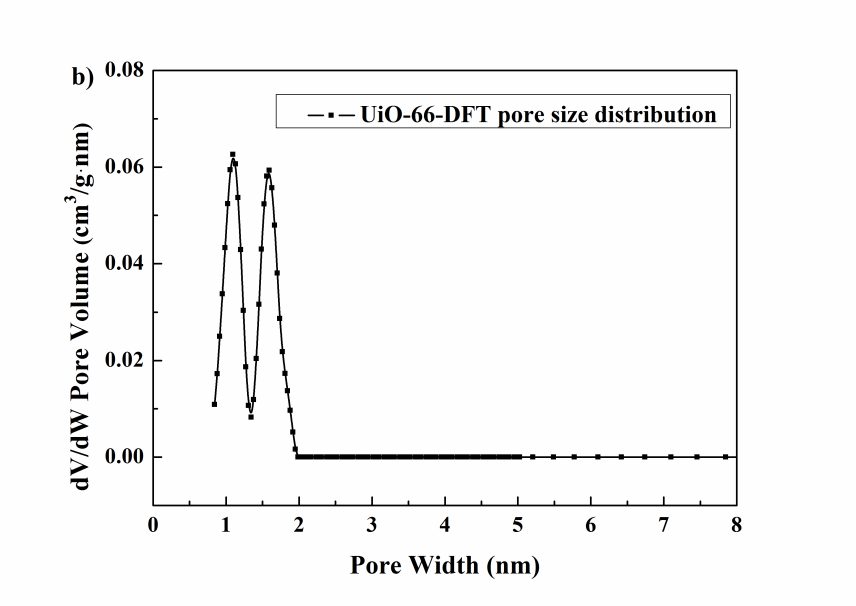


Fig. S5 a) N_2_ adsorption-desorption isotherm and b) NLDFT-pore size distribution of UiO-66.

Table S5. The pore structure data of UiO-66.

| Sample | S_BET_ (m^2^/g) | Pore volume (cm^3^/g) | Pore size(nm) |
| --- | --- | --- | --- |
| UiO-66 | 1076 | 1.06 | 6.7 |


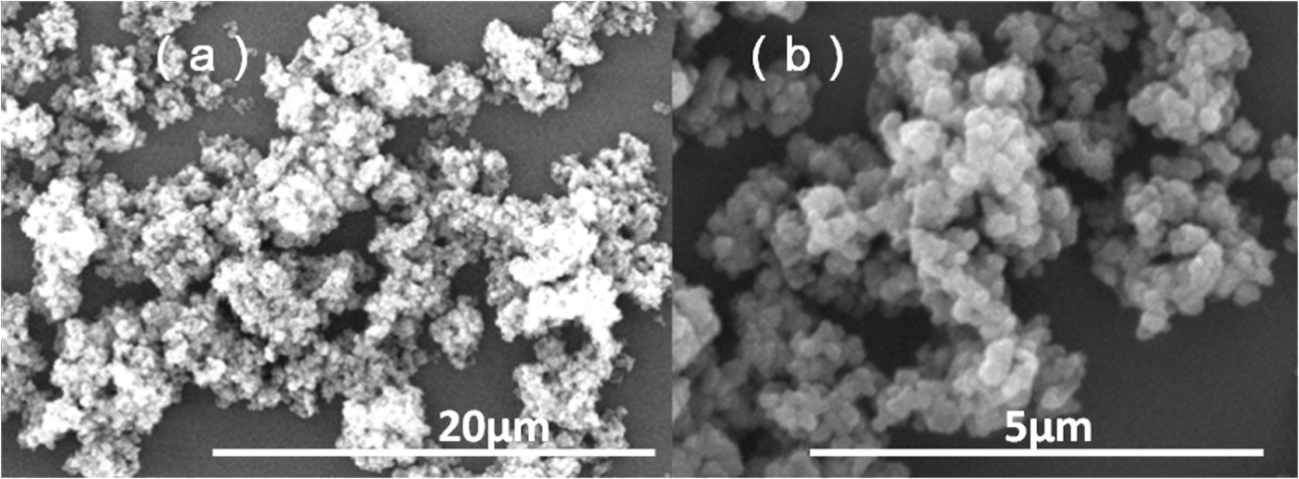


Fig. S6 SEM images of UiO-66.


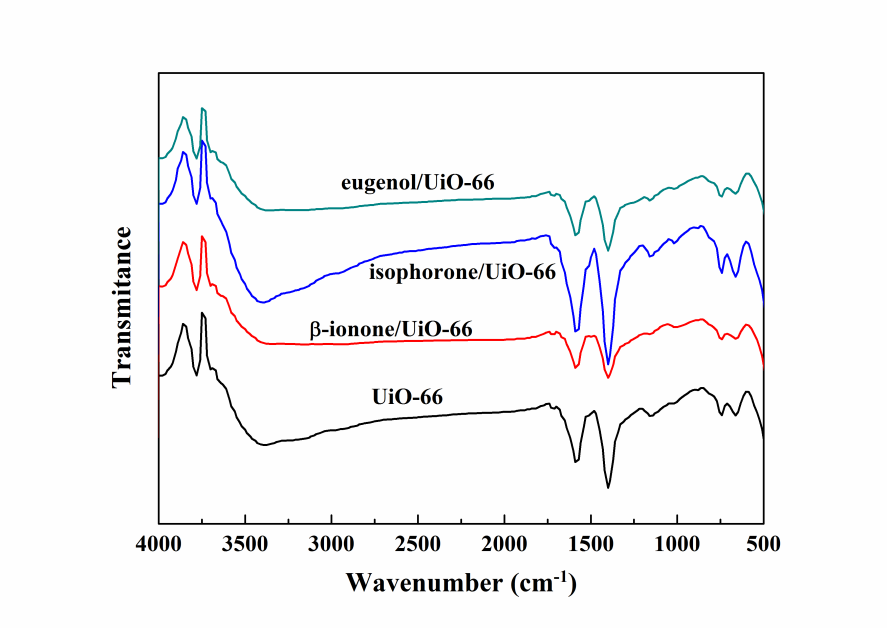


Fig.S7 Fourier transform infrared spectra for the empty UiO-66(Zr) and fragrances containing UiO-66(Zr).


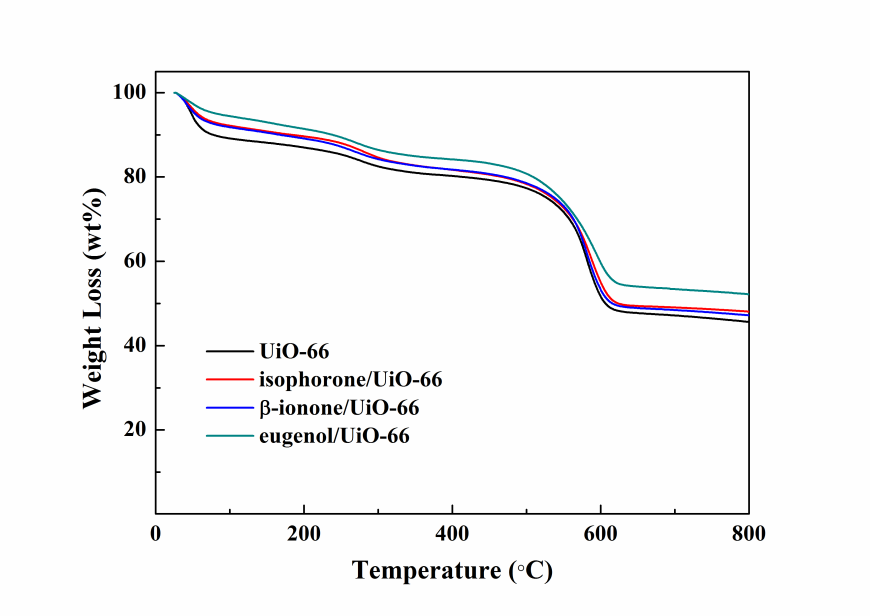


Fig.S8 Thermal Gravimetric Analysis plots of empty UiO-66(Zr) and fragrances containing UiO-66(Zr).
